# Fluvoxamine may prevent onset of psychosis: a case report of a patient at ultra-high risk of psychotic disorder

**DOI:** 10.1186/1744-859X-10-26

**Published:** 2011-09-30

**Authors:** Shigenori Tadokoro, Nobuhisa Kanahara, Shuichi Kikuchi, Kenji Hashimoto, Iyo Masaomi

**Affiliations:** 1 Department of Psychiatry, Chiba University Graduate School of Medicine, Chiba, Japan; 2 Department of Psychiatry, Sodegaura Satsukidai Hospital, Chiba, Japan; 3 Division of Clinical Neuroscience, Chiba University Center for Forensic Mental Health, Chiba, Japan

## Abstract

**Background:**

There is emerging evidence that antidepressants may be effective in preventing patients with non-specific and psychotic-like prodromal symptoms, defined as patients at ultra-high risk (UHR) of psychotic disorder, from transitioning to psychosis. However, the mechanism of such an effect is still unknown.

**Methods:**

We report the case of a 19-year-old Japanese man determined to be at UHR of psychotic disorder in whom fluvoxamine (one of the antidepressants with sigma-1 receptor agonism) showed preventive effects on psychotic-like prodromal symptoms.

**Results:**

Our patient's depressive symptoms were reduced and maintained below remission as a result of treatment with 100 mg/day of fluvoxamine. In addition, it is likely that an additional dose of fluvoxamine (50 mg/day) improved his psychotic-like prodromal symptoms directly, independent of its antidepressive effects.

**Conclusion:**

Fluvoxamine, a sigma-1 receptor agonist, may be effective in preventing patients at UHR of psychotic disorder from onset of psychosis via its neuroprotective/neurotropic actions, independent of its antidepressive effects.

## Background

In the past decade there has been increasing interest in the potential benefit of early pharmacological intervention in psychotic disorders. Patients with psychotic disorders show non-specific and psychotic-like prodromal symptoms preceding the onset of frank psychosis. Interestingly, there is emerging evidence that antidepressants may be effective in preventing patients who are at ultra-high risk (UHR) of psychotic disorder from transitioning to psychosis [1]. However, it is still unknown whether such prevention of psychosis onset by antidepressants is due to their antidepressive effects, such as mood improvement, or other pharmacological effects, such as neuroprotection. We present the case of a patient at UHR of psychotic disorder in which fluvoxamine, a selective serotonin reuptake inhibitor (SSRI) administered alone, prevented the onset of psychosis, independent of its antidepressive effects.

## Case presentation

The patient was a 19-year-old Japanese unmarried office worker with no personal or familial history of psychiatric problems. On presentation he complained of deterioration of his work performance. He had suffered from anhedonia, insomnia, loss of appetite, concentration deficit, and a sense of guilt for over 1 month, and was diagnosed with major depressive disorder according to *Diagnostic and Statistical Manual of Mental Disorders*, 4th Edition, Text Revision (DSM-IV-TR) criteria. His baseline score on the 17-item Hamilton Depression Rating Scale (HAM-D) was 23 points [2], and his Global Assessment of Functioning (GAF) score was 55 points [3]. Treatment with 50 mg/day of fluvoxamine was started. Then, 3 weeks later, his depressive symptoms improved and his fluvoxamine dose was increased to 100 mg/day. At 7 months after the start of treatment, his depressive symptoms disappeared (HAM-D score: 6). During the next 5 months, his quality of life (QoL) remained stable with satisfactory performance in his work (GAF score: 80).

About 1 year after the start of treatment, the patient's work performance deteriorated suddenly (GAF: 65) despite continuous treatment with 100 mg/day of fluvoxamine, without any concurrent symptoms of depression such as depressive mood or anhedonia. Then, 2 months later, he reported having a peculiar, highly confusing sensation that two work colleagues who were living in the same dormitory had spoken ill of him, unfairly accusing him of wearing unwashed clothes, among other things. In response, he had begun to delay his meal and bath times in order to shun contact with them, and to do his laundry several times a day. His QoL was highly disturbed and his work performance had worsened accordingly (GAF: 50). His disordered thoughts of persecution persisted for several months, and he was therefore diagnosed with UHR (attenuated psychosis group) under the criteria of the Comprehensive Assessment of At-Risk Mental States [4]. His fluvoxamine dose was increased to 125 mg/day. Then, 1 month later, he began to relax his efforts to avoid his colleagues and his QoL improved (GAF: 65). His fluvoxamine dose was further increased to 150 mg/day. At 1 month after that, he said brightly 'I don't care about them so much', and his QoL improved still further (GAF: 80). Treatment with 150 mg/day of fluvoxamine was maintained, and his QoL remained stable with satisfactory performance in his work, which lasted for more than 2 years of follow-up.

## Ethical approval

The treatment of the reported case was made according to standard clinical practice, and ethical approval was obtained from the Ethics Committee of Sodegaura Satsukidai Hospital, Japan.

## Discussion

To the best of our knowledge, this is the first report showing that fluvoxamine alone prevented a UHR patient from onset of psychosis. In this case, the patient's depressive symptoms were reduced and maintained below remission as a result of treatment with 100 mg/day of fluvoxamine. Therefore, it is estimated that the additional dose of fluvoxamine (50 mg/day) improved his psychotic-like prodromal symptoms directly, independent of its antidepressive effects.

Although the treatment with fluvoxamine modified the natural course of the case, psychotic-like symptoms never appeared during the major depressive episode. Furthermore, the patient's idea of persecution by his colleagues was odd and incongruent with his mood. Therefore, we estimated that both his psychotic-like symptoms and depressive symptoms were the prodromal symptoms of a UHR patient, rather than symptoms of psychotic major depression. Previously, Häfner *et al*. studied untreated psychotic and depressive symptoms retrospectively from onset until first admission, and demonstrated that delusional misinterpretations or delusional references were highly specific to schizophrenia (80.3% schizophrenia vs 6.2% unipolar depression), although the frequency of depressive symptoms showed surprising similarities between the two studied groups [5].

Although most antidepressants share blockade of serotonin transporters as a core mechanism, we reported that some antidepressants possess high to moderate affinity for the endoplasmic reticulum protein sigma-1 receptors [6], which are implicated in neuroprotection and neuronal plasticity [7-10]. Among all antidepressants, including SSRIs and typical tricyclic antidepressants, fluvoxamine has shown the most potent action at sigma-1 receptors, suggesting that sigma-1 receptors might be involved in fluvoxamine's mechanisms of action [6]. In cell culture systems, fluvoxamine, but not sertraline or paroxetine, stimulated nerve growth factor-induced neurite outgrowth in PC12 cells, and the effect of fluvoxamine was antagonized by treatment with the selective sigma-1 receptor antagonist NE-100 [11,12], suggesting that the agonism of fluvoxamine plays a role in neuronal plasticity.

We have previously hypothesized that the sigma-1 receptor agonist fluvoxamine might reduce the risk of subsequent transition to psychosis via its neuroprotective/neurotrophic actions [13]. To the best of our knowledge, this is the first case report supporting our hypothesis.

## Conclusions

This case suggests that fluvoxamine, a sigma-1 receptor agonist, may be effective in preventing patients at UHR of psychotic disorder from onset of psychosis. More detailed randomized, double-blind placebo-controlled studies using a large sample size will be needed to confirm this.

## Consent

Written informed consent was obtained from the patient for publication of this case report. A copy of the written consent is available for review by the Editor-in-Chief of this journal.

## Competing interests

The authors declare that they have no competing interests.

## Authors' contributions

ST, NK, and SK contributed to the clinical and rating evaluations during the follow-up periods. KH and MI conceived of the study and participated in its execution and coordination. All authors read and approved the final manuscript.
